# Assessment of selected psychosocial risk factors: stress, job burnout, and bullying in the case of medical staff as part of workplace ergonomics during the COVID-19 pandemic—A prospective pilot study

**DOI:** 10.3389/fpubh.2023.1169604

**Published:** 2023-05-04

**Authors:** Łukasz Rypicz, Izabela Witczak, Paweł Gawłowski, Hugh Pierre Salehi, Anna Kołcz

**Affiliations:** ^1^Division of Public Health, Department of Population Health, Faculty of Health Sciences, Wroclaw Medical University, Wrocław, Poland; ^2^Laboratory of Innovative Medical Education, Center for Medical Simulation, Wroclaw Medical University, Wrocław, Poland; ^3^Department of Engineering, The Ohio State University, Columbus, OH, United States; ^4^Ergonomics and Biomedical Monitoring Laboratory, Department of Physiotherapy, Faculty of Health Sciences, Wroclaw Medical University, Wrocław, Poland

**Keywords:** risk factors, medical staff, ergonomics, working condition, safety

## Abstract

**Background:**

The purpose of the pilot study conducted by the authors was to assess occupational risk in selected areas of psychosocial risk factors among health professions in a pilot study. Medical staff working in the healthcare sector experience stress, job burnout and bullying on a daily basis. Monitoring occupational risks in the above areas provides an opportunity to take appropriate preventive measures.

**Methods:**

The prospective online survey included 143 health care workers from various professional groups. Eighteen participants did not complete the survey, and the results of 125 participants were eventually included in the analysis. The study used health and safety questionnaires in the healthcare sector, which are not widely used as screening tools in Poland.

**Results:**

The following statistical methods were performed in the study: the Mann-Whitney test, Kruskal-Wallis test, Dunn's test. In addition, multivariate analysis was performed. The results obtained in the study indicate that the questionnaires used in the study can be widely used by employers or occupational medicine as screening tools.

**Conclusions:**

Our findings show that level of education attainment in healthcare is correlated with higher chance of experiencing stress and burnout. Among the surveyed professions, nurses reported a higher amount of stress and burnout. Paramedics reported the highest chance of being bullied at work. This can be explained by their nature of work which requires directly interacting with patients and their families. In addition, it should be noted that the tools used can be successfully applied in workplaces as elements of workplace ergonomics assessment in the context of cognitive ergonomics.

## 1. Introduction

Ergonomic research indicates that there is a cause-and-effect relationship between workplace ergonomics and the ability of staff to work. It appears that there are few studies from the healthcare sector that address ergonomic aspects of stress, job burnout or bullying (1, 2).

Healthcare workers during their daily work can experience excessive physical and mental exhaustion. This is associated with adverse consequences including an increased risk of developing mental health conditions like anxiety and depression. Negative impacts of burnout on mental health of healthcare workers have been highlighted in recent studies and the importance of maintaining a balance between personal life and work has been discussed. Additionally, evidence shows improving workplace ergonomics can improve psychosocial working conditions and prevent burnout (3).

Ergonomics deals with matching the needs of a job with the capabilities of the worker and the work environment to ensure the most efficient workplace while reducing the risk of injury (4). In addition, it is emphasized that ergonomics is gaining increasing recognition as an integral part of the system for ensuring fitness for work in the medical professions as well (5). It is increasingly noted that among the risk factors in the workplace are psychosocial factors, which play a significant role in ensuring a safe workplace. Psychosocial factors fall into the area of cognitive ergonomics, which includes perception, memory, reasoning and motor responses. They are extremely important because they affect interactions between people and other elements of the human-environment system (6–8).

One predictor of mental health among medical personnel is occupational burnout syndrome, which was defined in the 1970s by psychoanalyst Freudenberger (9–11). Occupational burnout is included in the 11th Revision of the International Classification of Diseases (ICD-11) as an occupational phenomenon—although it is not classified as a medical condition. It is defined as a conceptualized syndrome resulting from chronic workplace stress that has not been effectively managed. It is characterized by three dimensions: feelings of energy depletion or exhaustion, increased mental distance from one's job or feelings of negativity or cynicism about one's work, and decreased professional effectiveness (12). The scale of professional burnout is enormous. The results of studies conducted for years in the US indicate that professional burnout can affect up to 51% of doctors (13). Among nurses, the scale of the phenomenon is even greater, as globally professional burnout is said to be 15–60%, and in developed countries 49–57% (14, 15). Occupational burnout is strongly influenced by long-lasting stress levels, which among the medical profession are also very high (16).

Both stress and job burnout can influence the occurrence of bullying. Bullying in the workplace is a destructive phenomenon and disrupts the sense of security (17). It turns out that the phenomenon of bullying among medical professions is most prevalent in the professional group of nurses. They experience both verbal and physical violence (18). Rates of physical violence against doctors and nurses are 16.2 per 1,000 and 21.9 per 1,000, respectively. In the European Union, 52% of health care workers have experienced some type of aggression at work (19).

A review of the literature indicates that both occupational stress and burnout and bullying are common in the health care system. It is therefore important to monitor risk factors in this area. The authors attempted to assess occupational risk, additionally during the burden of the COVID-19 pandemic, which may have been an additional aggravating factor.

The purpose of the study conducted by the authors was to assess occupational risk in selected areas of psychosocial risk factors among health professions in a pilot study.

## 2. Materials and methods

### 2.1. Study design and setting

The prospective survey was conducted from November 1, 2021, to December 31, 2021, during the COVID-19 pandemic. The survey was conducted in an online format, using the electronic survey platform www.webankieta.pl. The survey is consisted of two parts:

a) Socio-demographic information of participants.
b) Participants' assessments of psychosocial risk factors.

The psychosocial risk factors section goes over three major themes and ach theme is consisted of 15 questions which are adopted from the European Commission's guide to health and safety risks in the healthcare sector [Europejska (20); the English-language version of the manual with questionnaires for each dimension of psychosocial factors can be found at the link: https://www.ilo.org/dyn/travail/docs/1965/osh.pdf]:

a) Workplace stresses;
b) Work related burnout;
c) Bullying at workplace.

Surveys chose either “applicable” or “not applicable” in response to psychosocial risk factors' questions. We used the aggregated scores to asses the severity of psychosocial risks. The risk levels were defined as follows:

a) no risk (1–5 marked answers “applicable”)—the need to take action on individual elements.
b) increased risk (6–10 marked “applicable” answers)—structural and control analyses are recommended.
c) high risk (11–15 marked “applicable” answers)—need for urgent structural and control analyses.

The survey was distributed to medical staff at the Wroclaw University of Medical Sciences including physicians, dentists, nurses, midwives, paramedics, and physiotherapists. Potential participants received a link to the survey through their medical social media groups. Participation in the survey was voluntary and data was collected anonymously. Participants could withdraw anytime. An IP address filtering (a numerical identifier given to a network interface) was used to avoid collecting duplicate responses from a participants.

After data collection was completed, a database was prepared and used in the statistical analysis.

### 2.2. Study population

The inclusion criterion for this study was active practice of a medical profession at the time of the survey, i.e., November–December 2021, during the COVID-19 pandemic. The study targeted 143 potential participants and 18 of them did not complete the survey. Uncomplete results were excluded from statistical analysis.

### 2.3. Ethical considerations

The study was carried out in accordance with the tenets of the Declaration of Helsinki and guidelines of Good Clinical Practice (21). Written information about the study was provided as an introduction to the survey, with an emphasis on the voluntary and anonymous nature of participation and its guaranteed confidentiality. By answering the questionnaire, participants gave their consent to participate in the study. The research project was approved by the Independent Bioethics Committee at the Wroclaw Medical University (No. KB−613/2021).

### 2.4. Statistical analyzes

Quantitative analysis was carried out by calculating the mean, standard deviation, median, and quartiles. Additionally, nominal variables were subjected to prevalence analysis based on the number and percentage of occurrences of each value. Comparison of the values of quantitative variables in the two groups was performed using the Mann-Whitney test. Comparison of the values of quantitative variables in three or more groups was performed using the Kruskal-Wallis test. When statistically significant differences were detected, *post-hoc* analysis was performed with Dunn's test to identify statistically significantly different groups. Multivariate analysis of the effect of multiple variables on a quantitative variable was performed using linear regression. The results are presented in the form of regression model parameter values with 95% confidence intervals. The analysis assumed a significance level of 0.05. So, all *p*-values below 0.05 were interpreted as indicating significant relationships. The analysis was performed in R software, version 4.2.2 (22).

## 3. Results

### 3.1. Single factor analysis

The characteristics of the study group with detailed socio-demographic data are presented in Table 1. A general summary of the level of risk identified in the three areas studied is presented in Table 2. Based on the results of Table 2, it should be noted most of the participants reported a high risk in all three areas, experiencing stress, burnout syndromes, and bullying- 63.2, 65.6, and 50.4%, respectively. These results are very disturbing, considering that the average age of the respondents was 32.1 years (Me = 30), and more than half of the respondents (57.6%) described their length of service as between 1 and 5 years. These shows relatively young people, at the beginning of their careers, experiencing high levels of risk from the group of psychosocial factors.

**Table 1:** Characteristics of the study group.

**Parameter**	**Total (*****N*** = **125)**	
Sex	Female	68 (54.40%)
	Male	57 (45.60%)
Age (years)	Mean (SD)	32.11 (7.65)
	Median (quartiles)	30 (26–36)
	Range	23–60
Marital status	Single	34 (27.20%)
	In relation to	91 (72.80%)
Residence	Country	27 (21.60%)
	City up to 50,000 inhabitants.	18 (14.40%)
	City of 50,000–150,000 inhabitants.	16 (12.80%)
	City of 150,000–500,000 inhabitants.	23 (18.40%)
	City with more than 500,000 inhabitants.	41 (32.80%)
Occupational group	Physiotherapist	5 (4.00%)
	Physician/dentist	24 (19.20%)
	Nurse	39 (31.20%)
	Midwife	5 (4.00%)
	Paramedic	51 (40.80%)
	Other	1 (0.80%)
Education	Secondary education	6 (4.80%)
	Bachelor's degree	44 (35.20%)
	Master's degree/medical doctor/dentist	68 (54.40%)
	PhD	7 (5.60%)
Seniority	Less than a year	6 (4.80%)
	1–5 years	72 (57.60%)
	6–10 years	19 (15.20%)
	11–15 years	12 (9.60%)
	16–20 years	7 (5.60%)
	More than 20 years	9 (7.20%)
Weekly working hours	20–39 h	18 (14.40%)
	40–59 h	57 (45.60%)
	60–79 h	38 (30.40%)
	80–99 h	9 (7.20%)
	100 h and more	3 (2.40%)
Place of employment	Hospital	86 (68.80%)
	Long-term care facilities	2 (1.60%)
	Primary health care	1 (0.80%)
	Others	36 (28.80%)
Works in shifts	No	24 (19.20%)
	Yes	101 (80.80%)
Type of ward	Surgical ward	46 (36.80%)
	Non-surgical ward	25 (20.00%)
	Not applicable	54 (43.20%)
Working in more than one place	No	53 (42.40%)
	Yes	72 (57.60%)

**Table 2:** Risk level results for each area.

**Risk area**	**Risk level**		
	**No risk**	**Increased risk**	**High risk**
Stress	9 (7.20%)	37 (29.60%)	79 (63.20%)
Burnout syndrome	6 (4.80%)	37 (29.60%)	82 (65.60%)
Bullying	12 (9.60%)	50 (40.00%)	63 (50.40%)

After determining the overall level of risk, a detailed analysis of socio-demographic data in correlation with the studied areas of psychosocial factors was performed. Those socio-demographic parameters with statistically significant differences (*p* < 0.05) were analyzed in detail. Among others, the level of education was included in the analysis (Table 3), and it was revealed that the risk in the area of stress is significantly higher in those with a bachelor's or master's degree than in those with a high school education. In addition, the risk in the area of burnout is significantly higher in those with a bachelor's or master's degree than in those with a high school education. It should be noted that in the case of the correlation of stress and level of education, this applies mainly to the nurses. The reasons for such a correlation can be explained by the number of duties and managerial activities. Nurses with higher education very often have professional roles with greater responsibilities. Therefore, the risk of burnout among those healthcare workers may be higher. Interestingly, the risk in the area of stress is significantly higher among those with work experience of 6–10 years than other groups (Table 4). In contrast, the risk in the area of burnout is significantly lower among the group working 20–39 h/week than in the other groups (Table 5). It can conclude that standard working hours (i.e., about 40 h/week) are the most optimal, and that overtime/additional employment can result in job burnout. This is explained further in the following in the next analysis—in terms of risk in the area of stress, burnout, bullying, which is significantly higher among those who work in multiple positions (Table 6).

**Table 3:** Influence of education level on the incidence of stress risk, occupational burnout syndrome, and bullying.

**Risk area**	**Education**	***N***	**Mean**	**SD**	**Median**	**Min**	**Max**	***Q*1**	***Q*3**	***p***
Stress	Secondary education—A	6	7.17	2.86	6.0	4	12	6.00	8.25	*p* = 0.049^*^
	Bachelor's degree—B	44	11.05	2.72	12.0	2	15	10.00	13.00	B, C>A
	Master's degree/medical doctor/dentist—C	68	10.94	3.02	12.0	3	15	10.00	13.00	
	PhD—D	7	9.57	3.26	8.0	6	13	7.00	13.00	
Burnout syndrome	Secondary education—A	6	8.17	3.37	9.0	3	13	6.75	9.00	*p* = 0.044^*^
	Bachelor's degree—B	44	11.64	2.86	12.0	4	15	9.75	14.00	C, B>A
	Master's degree/medical doctor/dentist—C	68	11.81	2.78	13.0	3	15	10.75	14.00	
	PhD—D	7	11.00	2.65	12.0	6	14	10.00	12.50	
Bullying	Secondary education	6	7.50	3.89	8.0	3	11	4.25	11.00	*p* = 0.303
	Bachelor's degree	44	10.64	2.82	11.0	3	15	8.75	13.00	
	Master's degree/medical doctor/dentist—C	68	10.26	3.41	10.5	3	15	7.75	13.00	
	PhD	7	10.29	2.14	10.0	8	13	8.50	12.00	

p—Kruskal-Wallis test + *post-hoc* analysis (Dunn's test), SD, standard deviation; Q1, lower quartile; Q3, upper quartile.

^*^Statistically significant difference (*p* < 0.05).

**Table 4:** The impact of seniority on the incidence of stress risk, burnout syndrome, and bullying.

**Risk area**	**Seniority**	***N***	**Mean**	**SD**	**Median**	**Min**	**Max**	***Q*1**	***Q*3**	***p***
Stress	Less than a year—A	6	8.50	4.72	9.0	3	14	4.50	12.00	*p* = 0.008^*^
	1–5 years—B	72	10.38	3.04	11.0	2	15	10.00	12.00	C>E, D, B, F, A
	6–10 years—C	19	12.79	1.87	13.0	7	15	12.00	14.00	
	11–15 years—D	12	10.83	2.55	11.5	7	15	8.75	12.25	
	16–20 years—E	7	11.14	1.07	12.0	10	12	10.00	12.00	
	More than 20 years—F	9	10.11	3.41	11.0	6	14	6.00	13.00	
Burnout syndrome	Less than a year	6	10.00	4.10	9.0	6	15	6.75	13.50	*p* = 0.278
	1–5 years	72	11.38	3.13	12.0	3	15	10.00	14.00	
	6–10 years	19	12.68	2.00	14.0	10	15	10.00	14.00	
	11–15 years	12	12.08	2.39	13.0	6	14	11.50	14.00	
	16–20 years	7	11.43	1.72	12.0	9	13	10.50	12.50	
	More than 20 years	9	10.67	2.74	10.0	6	14	9.00	13.00	
Bullying	Less than a year	6	9.33	3.67	10.0	5	14	6.25	11.50	*p* = 0.862
	1–5 years	72	10.31	3.11	11.0	3	15	8.00	13.00	
	6–10 years	19	10.74	3.02	10.0	7	15	8.00	13.50	
	11–15 years	12	10.75	3.55	12.0	3	15	8.00	13.00	
	16–20 years	7	9.86	3.93	10.0	5	15	7.00	12.50	
	More than 20 years	9	9.22	3.67	9.0	4	14	6.00	13.00	

p—Kruskal-Wallis test + *post-hoc* analysis (Dunn's test), SD, standard deviation; Q1, lower quartile; Q3, upper quartile.

^*^Statistically significant difference (*p* < 0.05).

**Table 5:** The impact of weekly working hours on the incidence of stress risk, burnout syndrome, and bullying.

**Risk area**	**Weekly working hours**	***N***	**Mean**	**SD**	**Median**	**Min**	**Max**	***Q*1**	***Q*3**	***p***
Stress	20–39 h	18	8.72	3.77	10.0	3	14	5.25	12.00	*p* = 0.069
	40–59 h	57	11.23	2.71	12.0	2	15	11.00	13.00	
	60–79 h	38	10.97	2.38	11.0	6	15	10.00	12.75	
	80 h and more	12	10.50	3.99	11.5	4	15	6.00	13.50	
Burnout syndrome	20–39 h—A	18	9.22	3.57	9.0	3	15	6.00	12.75	*p* = 0.013^*^
	40–59 h—B	57	11.77	2.56	12.0	3	15	10.00	14.00	C, D, B>A
	60–79 h—C	38	12.13	2.42	13.0	4	15	10.25	14.00	
	80 h and more—D	12	11.92	3.42	13.0	3	15	11.25	14.00	
Bullying	20–39 h	18	9.61	3.85	10.5	4	15	6.00	13.00	*p* = 0.749
	40–59 h	57	10.47	3.13	10.0	3	15	8.00	13.00	
	60–79 h	38	10.45	3.12	11.0	3	15	8.00	13.00	
	80 h and more	12	9.67	3.03	9.0	3	14	8.75	11.50	

p—Kruskal-Wallis test + *post-hoc* analysis (Dunn's test), SD, standard deviation; Q1, lower quartile; Q3, upper quartile.

^*^Statistically significant difference (*p* < 0.05).

**Table 6:** The impact of working more than one job on the incidence of stress risk, burnout syndrome, and bullying.

**Risk area**	**Working in more than one place**	***N***	**Mean**	**SD**	**Median**	**Min**	**Max**	***Q*1**	***Q*3**	***p***
Stress	No	53	9.91	3.48	11	2	15	7.00	13	*p* = 0.044^*^
	Yes	72	11.32	2.47	12	4	15	10.00	13	
Burnout syndrome	No	53	10.91	2.98	11	3	15	9.00	13	*p* = 0.019^*^
	Yes	72	11.99	2.77	13	3	15	10.75	14	
Bullying	No	53	9.58	3.22	10	3	15	7.00	12	*p* = 0.042^*^
	Yes	72	10.76	3.13	12	3	15	8.00	13	

p—Mann-Whitney test, SD, standard deviation; Q1, lower quartile; Q3, upper quartile.

^*^Statistically significant difference (*p* < 0.05).

In the areas discussed, the variable “gender” showed no statistically significant differences.

### 3.2. Multivariate analysis

The next step was multivariate analyses. Included in these analyses were those variables that had a significant effect on a given risk area in the univariate analyses or were close to significance (i.e., had *p* < 0.1), as well as occupational group, which is the main variable in this analysis.

#### 3.2.1. Stress

A multivariate linear regression model showed that significant (*p* > 0.05) independent predictors of risk in the area of stress are (Table 7):

a) Bachelor's degree: the regression parameter is 4.53, so it raises the risk by an average of 4.53 points relative to high school education.
b) Master's degree/doctor/dentist: The regression parameter is 4.91, so it raises the risk by an average of 4.91 points relative to secondary education.
c) Work experience of 6–10 years: the regression parameter is 3.17, so it raises the risk by an average of 3.17 points relative to < 1 year's experience.
d) Weekly working hours of 40–59 h: the regression parameter is 2.13, so it raises the risk by 2.13 points on average relative to working < 40 h/week.
e) Weekly working hours of 80 h or more: the regression parameter is 2.15, so it raises the risk by an average of 2.15 points relative to working < 40 h/week.
f) Working in more than one place: The regression parameter is 1.10, so it raises the risk by 1.10 points on average.

**Table 7:** Multivariate analysis—stress area.

**Feature**		**Parameter**	**95% CI**		***p***
Occupational group	Nurse/midwife	Ref.			
	Physician/dentist	−0.484	−1.865	0.897	0.494
	Paramedic	1.078	−0.136	2.292	0.085
	Other	0.661	−1.84	3.162	0.606
Residence	Country	Ref.			
	City up to 50,000 inhabitants	−1.174	−2.789	0.441	0.157
	City of 50,000–150,000 inhabitants	−0.457	−2.171	1.258	0.603
	City of 150,000–500,000 inhabitants	−0.929	−2.487	0.629	0.245
	City with more than 500,000 inhabitants	1.195	−0.168	2.558	0.089
Education	Secondary education	Ref.			
	Bachelor's degree	4.53	2.062	6.998	< 0.001^*^
	Master's degree/medical doctor/dentist	4.911	2.338	7.484	< 0.001^*^
	PhD	0.926	−2.199	4.051	0.563
Seniority	Less than a year	Ref.			
	1–5 years	1.14	−1.19	3.469	0.34
	6–10 years	3.176	0.542	5.81	0.02^*^
	11–15 years	1.932	−0.861	4.725	0.178
	16–20 years	2.121	−1.14	5.382	0.205
	More than 20 years	2.967	−0.131	6.065	0.063
Weekly working hours	20–39 h	Ref.			
	40–59 h	2.133	0.656	3.609	0.006^*^
	60–79 h	1.286	−0.304	2.876	0.116
	80 h and more	2.151	0.037	4.265	0.049^*^
Working in more than one place	No	Ref.			
	Yes	1.101	0.108	2.093	0.032^*^

p—multivariate linear regression.

^*^Relationship statistically significant (*p* < 0.05).

The correlations shown in the stress dimension that relate to educational level may be due to the fact that the vast majority of people have higher education. There are still nurses working in the health care system who have graduated from specialized schools—medical high schools. Medical personnel with master's degrees are more likely than those with bachelor's degrees to hold management positions, which further translates into higher stress levels. In turn, the weekly working hours—the greater, the higher the stress level is also a result of the fact that medical personnel often work in more than one place. Such behavior can determine a significant mental as well as physical burden. It should also be noted that the study was conducted during the pandemic period, when there was a shortage of staff, people worked beyond the norm to provide medical care to the needy.

#### 3.2.2. Professional burnout

In terms of burnout, a multivariate linear regression model showed that significant (*p* > 0.05) independent predictors of risk in this area are (Table 8):

a) Bachelor's degree: the regression parameter is 4.42, so it raises the risk by an average of 4.42 points relative to high school education.
b) Master's degree/doctor/dentist: The regression parameter is 5.05, so it raises the risk by an average of 5.05 points relative to secondary education.
c) Doctoral degree: The regression parameter is 3.59, so it raises the risk by an average of 3.59 points relative to secondary education.
d) Weekly working hours of 40–59 h: the regression parameter is 1.84, so it raises the risk by 1.84 points on average relative to working < 40 h/week.
e) Weekly working hours 60–79 h: The regression parameter is 2.21, so it raises the risk by an average of 2.21 points relative to working < 40 h/week.
f) Weekly working hours of 80 h or more: the regression parameter is 3.18, so it raises the risk by an average of 3.18 points relative to working < 40 h/week.
g) Place of employment other than a hospital: the regression parameter is −1.44, so it lowers the risk by an average of 1.44 points relative to hospital employment.

**Table 8:** Multivariate analysis–area of professional burnout.

**Feature**		**Parameter**	**95%CI**		***p***
Occupational group	Nurse/Midwife	Ref.			
	Physician/dentist	−0.423	−1.822	0.976	0.554
	Paramedic	1.067	−0.317	2.451	0.134
	Other	0.977	−1.594	3.549	0.458
Education	Secondary education	Ref.			
	Bachelor's degree	4.424	1.935	6.912	0.001^*^
	Master's degree/medical doctor/dentist	5.058	2.561	7.554	< 0.001^*^
	PhD	3.59	0.411	6.768	0.029^*^
Tygodniowy czas pracy	20–39 h	Ref.			
	40–59 h	1.841	0.313	3.368	0.02^*^
	60–79 h	2.211	0.643	3.779	0.007^*^
	80 h and more	3.184	1.107	5.261	0.003^*^
Place of employment	Hospital	Ref.			
	Other	−1.442	−2.824	−0.06	0.043^*^
Working in more than one place	No	Ref.			
	Yes	0.818	−0.173	1.809	0.108

p—multivariate linear regression.

^*^Relationship statistically significant (*p* < 0.05).

The problem of burnout among medical staff is widely studied. It is influenced by several factors: medics work too much (more than 40 h a week, in more than one place), we have a shortage of medical personnel (significant workload, rationing of care) and there is a lack of prevention in this area. Employers do not take measures to counteract professional burnout, and prc medicine does not give this problem the attention it deserves.

#### 3.2.3. Bullying

In terms of bullying, a multivariate linear regression model showed that significant (*p* > 0.05) independent predictors of risk in this area are (Table 9):

a) Practicing as a paramedic: The regression parameter is 2.11, so it raises the risk by an average of 2.11 points relative to the nursing/midwifery profession.
b) Working in more than one place: The regression parameter is 1.39, so it raises the risk by 1.39 points on average.

**Table 9:** Multivariate analysis—the area of bullying.

**Feature**		**Parameter**	**95%CI**		***p***
Occupational group	Nurse/midwife	Ref.			
	Physician/dentist	−0.399	−2.057	1.258	0.638
	Paramedic	2.117	0.441	3.793	0.015^*^
	Other	0.392	−2.576	3.36	0.796
Sex	Female	Ref.			
	Male	−0.941	−2.379	0.497	0.202
Seniority	Less than a year	Ref.			
	1–5 years	0.762	−2.021	3.546	0.592
	6–10 years	1.305	−1.755	4.365	0.405
	11–15 years	0.879	−2.354	4.111	0.595
	16–20 years	1.484	−2.439	5.407	0.46
	More than 20 years	−0.468	−3.88	2.944	0.788
Place of employment	Hospital	Ref.			
	Other	−1.103	−3.142	0.937	0.292
Type of ward	Surgical ward	Ref.			
	Non-surgical ward	1.591	−0.046	3.227	0.059
	Not applicable	−0.356	−2.322	1.61	0.723
Working in more than one place	No	Ref.			
	Yes	1.395	0.201	2.589	0.024^*^

p—multivariate linear regression.

^*^Relationship statistically significant (*p* < 0.05).

Experiencing violence by medical personnel, especially during a pandemic, was not unusual. It was related to fear of the SARS-CoV-2 virus, the consequences of COVID-19 disease or fear for the health of their loved ones. People in highly stressful situations behave irrationally which may be related to the results of the study. It should be noted that medical personnel very often experience violence—both psychological (such as verbal) and physical. Paramedics, are the people who are on the front line at accidents or in hospital emergency departments. They often have to deal with patients who are under the influence of psychoactive substances, which can potentiate aggressive behavior.

## 4. Discussion

Medical staff are an essential part of the healthcare system, without them the provision of medical care is impossible. A safe and healthy workplace is critical to maintaining the mental health of healthcare workers. It is the resultant of ergonomic conditions and principles in the workplace. Ensuring the above is not possible without monitoring occupational risks in selected areas. The authors of the minor paper decided to focus on selected factors from the area of psychosocial factors, and the study was carried out during the period of increased tension, stress or fear caused by the COVID-19 pandemic.

The results revealed a significant problem likely associated with the absence of coping strategies for psychosocial risk factors. With respect to stress, burnout, and bullying, over half of respondents were in the high-risk group −63.2, 65.6, and 50.4% of respondents, respectively.

Our research shows that burnout is common amongst health care workers treating patients with COVID-19. Age, gender, category of employment and place of practice contribute to the level of employee burnout (23). The study found that medical staff with higher levels of educational attainment are more likely to suffer from burnout syndrome than those with high school education.

Sirilla (24) showed that the level of burnout recorded in oncology nurses was inversely proportional to the level of education—the higher the level of education, the lower the level of burnout. Grisales-Romero et al. (25) exhibited a similar relationship. Moreover, Lou et al. showed that during the COVID-19 pandemic, nurses experienced more stress than doctors (26).

Another factor which may increase the risk of stress or burnout is workload—in terms of hours. Second jobs can increase the risk to mental health. Stehman's work points out that working more than 40 h per week, being on call or working at night can greatly accelerate the burnout process (27).

Stress and burn-out can be linked to bullying, which can lead to verbal and physical abuse. The results of the multivariate analysis showed that practice in the paramedic profession and working in more than one location are associated with a higher risk of workplace bullying. Campo's study found that 46.6% of paramedics believe they have been verbally abused in the past year, and nearly 18% have reported being bullied which is a low percentage of total incidents (28).

European Agency for Safety and Health at Work (EU-OSHA) highlights psychosocial risks, which can result, for example, from poor work planning, poor work organization and management, and an unfavorable social work environment. Psychosocial hazards can lead to negative mental, physical and social effects, such as work-related stress, burnout or depression (29). The European Agency for Safety and Health at Work (EU-OSHA) has commissioned a Flash Eurobarometer survey in April 2022 to obtain more information on the state of OSH in post-pandemic workplaces, including psychosocial risk factors. EU-OSHA has commissioned a Flash Eurobarometer survey in April 2022 to obtain more information on the state of OSH in post-pandemic workplaces, including psychosocial risk factors. Respondents to the survey (46% of those surveyed) indicated that they are exposed to severe time pressure or work overload, with the experience of violence or verbal abuse from patients mentioned by 16% of respondents across the EU. Interestingly, employees from countries such as Finland, Malta, Sweden and Denmark were more likely than Poles to discuss their mental health with their employer. More than 4 in 10 respondents across the EU agree that their stress at work has increased as a result of the COVID-19 pandemic (30). The above findings correspond with the results obtained in this study. The pandemic has contributed to an increase in psychosocial burden among workers, including those in the healthcare sector.

## 5. Conclusion

Our findings show that level of education attainment in healthcare is correlated with higher chance of experiencing stress and burnout. Among the surveyed professions, nurses reported a higher amount of stress and burnout. Paramedics reported the highest chance of being bullied at work. This can be explained by their nature of work which requires directly interacting with patients and their families.

Considering the high rate of reported stress, burnout, and bullying among healthcare workers, it is important to increase awareness of the staff about psychosocial risk factors occupational risks. Considering the current shortage of human resources and increasing demand of aging population in west for healthcare related services, investing in educational programs for medical staff to make them familiar with strategies for managing occupational stress, burnout, and bullying can result in better less turnover of the staff, better mental health, and eventually better patient outcome. Additionally, investing in workplace management enhancement programs and improving ergonomics can prepare us for next potential pandemic and improve medical staff work satisfaction.

Practical implications for employers in the health care sector. A small pilot study has shown that questionnaires for assessing psychosocial risk factors in the areas of stress, occupational burnout and violence can be used in workplaces as screening tools for preventive measures against the mental health of health care workers. Based on the results obtained, corrective measures can be implemented in the areas of stress re-education, occupational burnout or violence prevention. Studies show that workplace ergonomics has a huge impact on the health of employees, and it is the employer's responsibility to provide safe working conditions. This is especially important at a time when the health care system is facing a major challenge—an increase in demand for medical care and a shortage of medical staff.

## Data availability statement

The raw data supporting the conclusions of this article will be made available by the authors, without undue reservation.

## Author contributions

ŁR and AK: conceptualization. ŁR: methodology, software, formal analysis, visualization, project administration, and supervision. ŁR and HS: validation. PG: investigation. ŁR and PG: resources. ŁR, IW, and HS: data curation and writing—original draft preparation. ŁR, AK, and HS: writing—review and editing. ŁR, IW, AK, and PG: funding acquisition. All authors contributed to the article and approved the submitted version.
